# Anxiety, depression and hyperventilation symptoms in treatment-resistant severe asthma

**DOI:** 10.1186/2045-7022-5-S2-P7

**Published:** 2015-03-23

**Authors:** Tan Li Leng karen, Tay Chee Kiang, Yii Anthony, Chan Kwok Wai Adrian, Lapperre Therese Sophie, Koh Mariko Siyue

**Affiliations:** 1 Singapore General Hospital, Department of Respiratory Medicine, Singapore, Singapore

## Background

Psychological morbidity is associated with frequent asthma exacerbations, higher health care utilization, as well as near-fatal and fatal asthma. The prevalence of psychological comorbidities in patients with severe asthma is not well-studied. The aim of our study was to evaluate the prevalence of anxiety, depression and hyperventilation, and correlate these symptoms with asthma control, exacerbation frequency and lung function in severe asthmatics.

## Methods

Patients who fulfilled the WHO definition of "treatment-resistant severe asthma" (at least combination high-dose inhaled corticosteroids and long-acting beta agonists) were included. Patients literate in English completed the Hospital Anxiety and Depression Scale (HADS) and Nijmegen Questionnaire during a regular doctor's visit at our Severe Asthma clinic. The HADS questionnaire comprises 7 anxiety and 7 depression questions. A cut-off score of ≥8 indicates a probable case of anxiety or depression. Nijmegen Questionnaire is a screening instrument for hyperventilation. A score of ≥23 suggests hyperventilation. Relation between these scores and Asthma Control Test, exacerbation frequency, health care utilization and lung function test results were analyzed.

## Results

52 patients were included in the analysis: 24 (46.2%) male, mean age 37.5 ±16.6 years, mean ACT score 16.8 ±4.5. The prevalence of anxiety was 44.2%, depression was 19.2% and hyperventilation was 28.8%. Patients with anxiety were more likely to have uncontrolled asthma (defined as Asthma Control Test score of 19 or less) compared to those without (87.0% vs. 51.7%, p=0.009). Similarly, patients with hyperventilation were more likely to have uncontrolled asthma compared to those without (93.3% vs. 56.8%, p=0.011). There was no association between depression and asthma control. Patients with anxiety had lower FEV_1_ % predicted compared to those without (69.4±17.2% vs. 81.2±21.2%, p=0.036). There was no association between depression or hyperventilation and FEV_1_. No association was found between anxiety, depression or hyperventilation and frequency of asthma exacerbations.

## Conclusion

A high prevalence of anxiety, hyperventilation and depression exists amongst our severe asthmatics. Hyperventilation and anxiety were associated with uncontrolled asthma, and presence of anxiety was associated with a lower FEV_1_ % predicted. This suggests that there is an objective association between anxiety and uncontrolled asthma, although the causal link remains unclear.

